# Mutational Landscape and Treatment Response in Extensive-Stage Small-Cell Lung Cancer: A Single-Center Real-World Analysis

**DOI:** 10.3390/curroncol33050256

**Published:** 2026-04-29

**Authors:** Meizeng Li, Lianying Guo, Ruiying Zhao, Shengnan Chen, Shengji Ma, Chan Xiang, Yuchen Han

**Affiliations:** Department of Pathology, Shanghai Chest Hospital, Shanghai Jiao Tong University School of Medicine, Shanghai 200025, China; meizenglee@shsmu.edu.cn (M.L.);

**Keywords:** chemoimmunotherapy, genomic profiling, adrenal metastasis, prognostic signatures, real-world evidence

## Abstract

**Objectives:** Small-cell lung cancer (SCLC) is an aggressive malignancy often diagnosed at the extensive stage (ES-SCLC). While chemoimmunotherapy (CIT) has emerged as a first-line option, SCLC’s “cold” immune profile limits broad efficacy. This study evaluates the real-world clinical efficacy of CIT versus chemotherapy (CT) alone and analyzes the association between gene mutation characteristics and clinical indicators. **Methods:** We retrospectively analyzed 170 patients with ES-SCLC treated at a single center between January 2020 and January 2024. Patients were categorized by first-line treatment (CIT vs. CT). Subgroup analyses were conducted to evaluate treatment response. Genomic profiling was integrated for a subset of patients to identify associations between mutation signatures and clinicopathological factors. **Results:** Of the 115 patients (67.6%) who received CIT and 55 (32.4%) who received CT, the CIT group achieved a significantly higher objective response rate (76.5% vs. 56.4%). Median progression-free survival was numerically but not significantly longer in the CIT group (6.0 vs. 5.8 months). Adrenal metastasis was identified as an independent adverse prognostic factor. Genomic analysis revealed site-specific correlations: *MYC* mutations with pleural metastasis, *NTRK3* with brain metastasis, *ALK* with adrenal metastasis, and *NTRK1* with intrapulmonary metastasis. Additionally, smokers showed higher mutation frequencies in *SMAD4* and *PIK3CA*. **Conclusions:** CIT significantly improves initial response rates in ES-SCLC compared to CT alone. Baseline adrenal metastasis serves as a poor prognostic indicator. Distinct genomic mutation signatures are associated with clinical characteristics, suggesting potential pathways for personalized treatment strategies.

## Simple Summary

Small-cell lung cancer is a highly aggressive form of lung cancer, especially when diagnosed at an advanced stage. While combining chemotherapy with immunotherapy has become a standard treatment, not all patients benefit equally. In this real-world study, we compared the effectiveness of chemoimmunotherapy versus chemotherapy alone in 170 patients and analyzed whether genetic mutations are linked to clinical features. We found that adding immunotherapy significantly improved the tumor objective response rate. Patients with adrenal gland metastasis had worse outcomes. We also identified specific gene mutations associated with metastasis to different organs (e.g., brain, pleura, adrenal). By linking genetic profiles to clinical outcomes, this study provides insights for developing personalized treatment strategies in SCLC.

## 1. Introduction

Small-cell lung cancer (SCLC) is an aggressive, smoking-related neuroendocrine malignancy that comprises about 15% of lung cancers. It typically demonstrates rapid progression and early dissemination, leading to a diagnosis of extensive-stage (ES) disease in approximately 70% of patients, as defined by the Veterans Administration Lung Study Group (VALG) staging system. This clinical presentation contributes to a very poor five-year survival rate. For decades, platinum-doublet chemotherapy has been the cornerstone of first-line treatment for ES-SCLC. While offering high initial response rates, its efficacy is notoriously transient due to the rapid emergence of multidrug resistance. Consequently, median progression-free survival (PFS) and overall survival (OS) have historically plateaued at 5–6 and 9–10 months, respectively [1]. This inevitable rapid relapse, coupled with the highly heterogeneous nature of the tumor, highlights a critical unmet clinical need for novel therapeutic strategies and predictive biomarkers to improve long-term outcomes in this devastating disease.

The incorporation of immune checkpoint inhibitors (ICIs) into first-line regimens marks a significant recent therapeutic shift. Landmark phase III clinical trials such as IMpower133 and CASPIAN demonstrated that adding a PD-L1 inhibitor—specifically atezolizumab or durvalumab—to representative platinum–etoposide chemotherapy significantly improved median PFS and OS compared to chemotherapy alone [2,3]. Based on these pivotal studies, chemoimmunotherapy (CIT) has become the new standard of care for ES-SCLC. Nevertheless, the absolute survival benefit from immunotherapy remains modest, and long-term outcomes for most patients are still unfavorable. Furthermore, clinical trials enforce strict enrollment criteria, frequently excluding patients with poor performance status, untreated brain metastases, or complex comorbidities. Therefore, understanding the true effectiveness of CIT versus traditional chemotherapy in more diverse, representative clinical settings is urgently needed, making real-world evaluations indispensable [4,5]. Leveraging real-world data, this study systematically characterized and compared the efficacy of CIT versus standard chemotherapy in ES-SCLC patients, while performing a comprehensive evaluation of baseline clinicopathological features. Because the therapeutic landscape for ES-SCLC is hindered by high genomic heterogeneity and a lack of reliable predictive biomarkers, we also delineated the genomic landscape and co-mutation patterns in SCLC. By investigating their correlations with clinical phenotypes and treatment responses, this study aims to provide critical insights into disease prognosis and facilitate the development of more personalized and improved therapeutic approaches.

## 2. Patients and Methods

### 2.1. Patients

**Figure 1:**
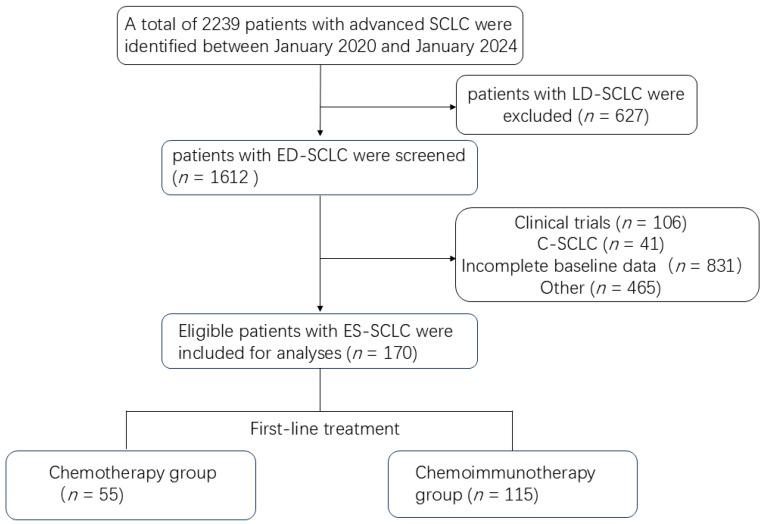
Flowchart of the study. LS-SCLC, limited-stage small-cell lung cancer; ES-SCLC, extensive-stage small-cell lung cancer; C-SCLC, combined small-cell lung carcinoma.

This was a single-center, retrospective cohort study. Data were collected from patients diagnosed and treated for ES-SCLC at Shanghai Chest Hospital between January 2020 and January 2024. The inclusion criteria were as follows: (1) all SCLC cases were diagnosed per WHO criteria using immunohistochemistry for neuroendocrine (CD56, SYN, ChgA, INSM1), epithelial (CK), and proliferative (Ki-67) markers, with TTF-1 supporting pulmonary origin; (2) histologically and radiologically confirmed SCLC with at least one measurable lesion; (3) classified as ES-SCLC according to the Veterans Administration Lung Study Group (VALG) staging system; and (4) received first-line treatment with either chemotherapy or chemoimmunotherapy and completed at least two cycles (21 days per cycle) of therapy. Patients were excluded for any of the following: (1) incomplete demographic or treatment data; (2) a history of other concurrent malignant tumors; or (3) receipt of investigational drugs. A total of 170 patients who met all the criteria were ultimately enrolled in the final cohort. The patient screening process and outcomes are detailed in Figure 1.

### 2.2. Data Collection and Assessment

Patients were stratified into two cohorts based on the inclusion of immunotherapy in their first-line treatment: the CIT and the CT. Collected data encompassed demographic and baseline clinical characteristics, including sex, age, smoking history, anatomical location of the primary tumor, disease stage and metastatic sites at diagnosis, PD-L1 expression level, immunohistochemistry gene mutation status, and PFS. The chemotherapeutic agents used primarily included etoposide, carboplatin, and nedaplatin. The immune checkpoint inhibitors involved comprised PD-L1 inhibitors (atezolizumab, durvalumab) and PD-1 inhibitors (pembrolizumab, tislelizumab, toripalimab, and camrelizumab).

This study defined PFS as the primary endpoint, calculated from the commencement of first-line therapy until radiologically confirmed disease progression or death from any cause. The data cutoff for PFS and survival follow-up was 30 July 2024. Secondary endpoints comprised the objective response rate (ORR) and disease control rate (DCR), both determined by the investigators based on Response Evaluation Criteria in Solid Tumors (RECIST 1.1) guidelines. Specifically, ORR was defined as the combined incidence of complete response (CR) and partial response (PR). DCR was defined as the proportion of patients exhibiting a best objective response of CR, PR, or stable disease (SD).

### 2.3. Statistical Analysis

All statistical analyses in this study were performed using SPSS software (version 25.0). Differences in continuous variables were evaluated using a *t*-test or the Wilcoxon rank-sum test. Categorical variables were compared using the chi-square test or Fisher’s exact test, as appropriate. Univariate and multivariate analyses were conducted using the Cox proportional hazards regression model to evaluate the association between various variables and PFS, with results presented as hazard ratios (HRs) and their 95% confidence intervals (CIs). Survival curves were generated using the Kaplan–Meier method and compared with the log-rank test. All tests were two-sided, and a *p* value < 0.05 was considered statistically significant.

### 2.4. Ethics Statement

Prior to initiation, this study obtained formal approval from the Ethics Committee of Shanghai Chest Hospital. All subsequent procedures and data collection activities strictly adhered to the ethical standards and protocols authorized by the committee.

## 3. Results

### 3.1. Baseline Characteristics of ES-SCLC Patients

170 eligible ES-SCLC patients were identified from 2239 pathology records screened between January 2020 and January 2024. The baseline characteristics are presented in Table 1. The enrolled population was predominantly male (139, 81.8%) and had a smoking history (114, 67.1%), with central (123, 72.4%) and stage IV (124, 72.9%) tumors being most common. At initial diagnosis, metastases were frequently observed in the bone (55, 32.4%), liver (33, 19.4%), and brain (30, 17.6%); metastases to lymph nodes, adrenal glands, lungs, and pleura constituted 14%, 14%, 13%, and 12% of cases, respectively. Immunohistochemical analysis demonstrated high positive rates for CD56 (98.24%), cytokeratin (CK) (98.24%), TTF-1 (90.00%), synaptophysin (SYN) (95.65%), and INSM1 (83.08%). Conversely, chromogranin A (ChgA) positivity was low (13.66%). The proliferation marker Ki-67 showed an average positivity of 77.78% across all cases. The highest expression was 95%, and the lowest was 40%. For first-line treatment, 55 patients received EC/EP chemotherapy (CT group) and 115 received chemoimmunotherapy (CIT group). In the CIT group, most patients received a PD-L1 inhibitor (*n* = 97), while 16 received a PD-1 inhibitor; five patients in this group were administered two cycles of induction chemotherapy prior to CIT. The patient selection flowchart is shown in Figure 1.

### 3.2. Survival Analysis

**Figure 2:**
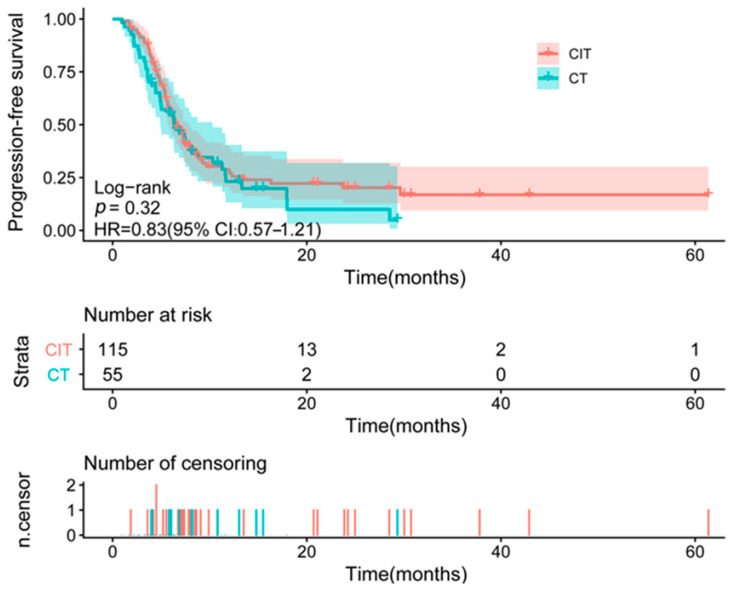
Kaplan–Meier curve for progression-free survival in ES-SCLC. HR, hazard ratio; CT, chemotherapy group; CIT, chemoimmunotherapy group.

After a median follow-up of 21.3 months (95% CI: 20.0–24.5), with assessments by CT scan every 2–3 months, 119 patients had experienced disease progression. The median PFS was 14.5 months (95% CI: 10.1–18.5) in the CT group (41/55 events) and 15.0 months (95% CI: 13.5–17.3) in the CIT group (78/115 events). The corresponding HR for progression or death was 0.83 (95% CI: 0.57–1.21) for CIT versus CT, which was not statistically significant (Figure 2).

Objective tumor responses are summarized in Table 2. In the CT group, PR, SD, and PD were observed in 31 (56.4%), 18 (32.7%), and 6 (10.9%) patients, respectively. Conversely, the CIT group demonstrated higher rates of PR in 88 patients (76.5%) and SD in 22 patients (19.1%), with only 5 (4.4%) experiencing PD. The confirmed ORR was significantly higher in the CIT group than in the CT group (76.5% [95% CI, 67.9–83.3] vs. 56.4% [95% CI, 43.3–68.6]; *p* = 0.007). The DCR also favored the CIT group (95.7% [95% CI, 90.2–98.1] vs. 89.1% [95% CI, 78.2–94.9]; *p* = 0.10).

Univariate Cox regression identified liver metastasis and adrenal metastasis as significant adverse prognostic factors for PFS (*p* < 0.05), while no significant associations were found for other clinical variables, including age, gender, smoking history, tumor location, disease stage, PD-L1 expression, and metastatic involvement in other common sites (Table 3). On multivariate analysis, adrenal metastasis was confirmed as an independent predictor of poorer PFS (Table 4).

### 3.3. The Mutational Landscape of ES-SCLC

**Figure 3:**
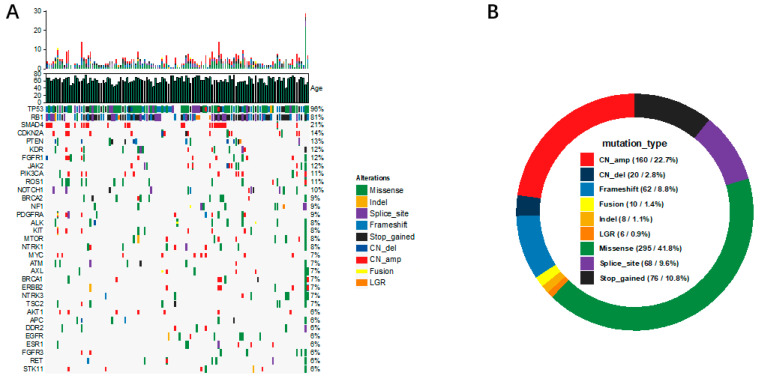
Mutational landscape of SCLC: (**A**) Genomic alteration profiling. Patterns of the 33 most frequent gene alterations identified in SCLC tumors. Genes are indicated on the left and their alteration frequency on the right. (**B**) Distribution of mutation types in SCLC.

Of the 170 enrolled patients, 121 had available next-generation sequencing data based on a 68-gene lung cancer panel. Further analysis was conducted on genes with a mutation frequency >5%. The dual inactivation of the tumor suppressor genes *RB1* and *TP53* constitutes the genetic cornerstone of SCLC pathogenesis. As shown in Figure 3A, *TP53* and *RB1* mutations were the most prevalent in our cohort, with mutation frequencies of 95.8% (116/121) and 80.9% (98/121), respectively, consistent with rates reported in previous literature [6]. Other recurrently mutated genes, in descending order of frequency, included *SMAD4* (21.4%, 26/121), *CDKN2A* (14.0%, 17/121), *PTEN* (13.2%, 16/121), *KDR* (12.3%, 15/121), *FGFR1* and *JAK2* (11.5%, 14/121), *PIK3CA* and *ROS1* (10.7%, 13/121), *NOTCH1* (9.9%, 12/121), *BRCA2*, *NF1*, and *PDGFRA* (9.0%, 11/121), as well as *ALK*, *KIT*, *MTOR*, and *NTRK1* (8.2%, 10/121). The spectrum of the most frequent genetic alterations comprised missense mutations (41.8%), amplifications (22.7%), stop-gain variants (10.8%), splice-site mutations (9.6%), and frameshift mutations (8.8%) (Figure 3B).

### 3.4. Co-Occurrence and Mutual Exclusivity of Somatic Alterations in SCLC

**Figure 4:**
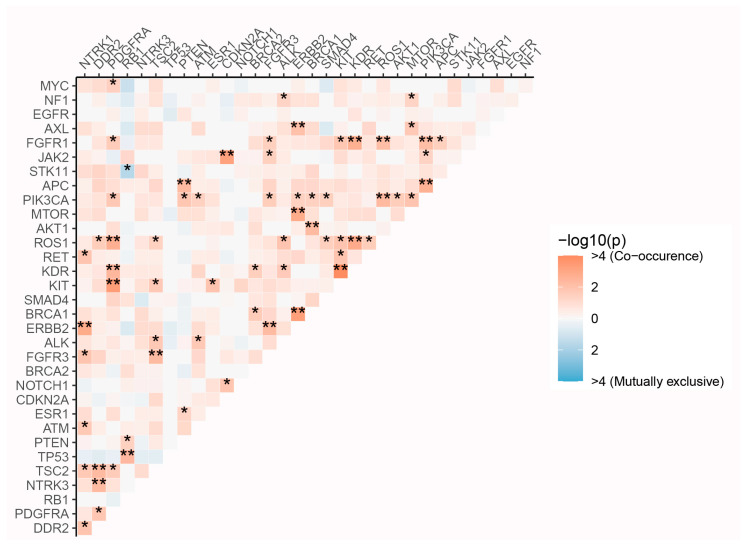
Co-occurrence and mutual exclusivity of genetic alterations in SCLC. * *p* < 0.05; ** *p* < 0.01.

In our subsequent analysis of co-occurring genomic alterations in SCLC, we identified several mutations that are significantly co-mutated, suggesting that these lesions cooperate to promote a malignant phenotype (Figure 4). For instance, our analysis confirmed the co-occurrence of *TP53* and *RB1* mutations; their simultaneous inactivation is known to lead to uncontrolled cell proliferation, thereby promoting SCLC development [7].

Notably, the significant co-mutated genes were not randomly distributed but were highly enriched within several core biological pathways. Primarily, synergistic activation of the PI3K/AKT/mTOR pathway was observed. *PIK3CA* acted as a central hub in the network, co-mutating with ten distinct genes, including upstream receptor tyrosine kinases (RTKs) such as *ERBB2* and *FGFR3*; other pathway members like *AKT1* and *MTOR*; the tumor suppressor *PTEN* (via inactivation); and DNA damage response (DDR) genes, including *ATM* and *BRCA1*. *PTEN*, a key negative regulator of the PI3K-AKT-mTOR signaling pathway, promotes transcriptional heterogeneity and can consequently drive resistance to immunotherapy upon its loss [8]. The concurrent presence of *PIK3CA* mutations and *PTEN* loss may represent a dual activation mechanism, enhancing PI3K/AKT/mTOR pathway activity and serving as a common driver of tumor progression [9,10].

Moreover, analysis revealed cross-talk between the DNA damage repair and RTK/PI3K signaling pathways, characterized by specific co-occurring genetic pairs: *PIK3CA* with both *ATM* and *BRCA1*, *AKT1* with *BRCA1*, and *ERBB2* with *ATM*. As the most commonly mutated DDR gene, the co-mutation of *ATM* with *PIK3CA* potentially indicates a link to increased genomic instability.

Our genomic profiling uncovered co-mutated *BRCA1* and *BRCA2*, pointing to a constitutive homologous recombination deficiency (HRD) in a subset of patients [11]. HRD is a well-validated predictive biomarker that confers heightened sensitivity to PARP inhibitors and can also forecast response to platinum-based chemotherapy [12].

Mutual exclusivity is a well-documented phenomenon in cancer genomics, where large-scale sequencing studies consistently show that driver oncogene mutations tend to be mutually exclusive, though the biological basis for this pattern is not fully elucidated [13]. We identified a striking pattern of mutual exclusivity between *STK11* and *RB1* mutations in SCLC. This finding is particularly significant given that *RB1* inactivation is a near-universal, defining feature of SCLC, while *STK11* (*LKB1*) loss—a known regulator of cell cycle and polarity—is well-documented in non-small-cell lung cancer (NSCLC) [14]. This novel observation may provide a clue for refining the molecular classification of SCLC. Overall, this work reveals a complex interactome in SCLC that extends beyond *TP53/RB1*.

The landscape of co-occurring and mutually exclusive genetic events highlighted here offers a valuable framework for elucidating pathogenic mechanisms and revealing new therapeutic opportunities.

### 3.5. Correlation of Somatic Mutations with Clinicopathologic Features in SCLC

**Figure 5:**
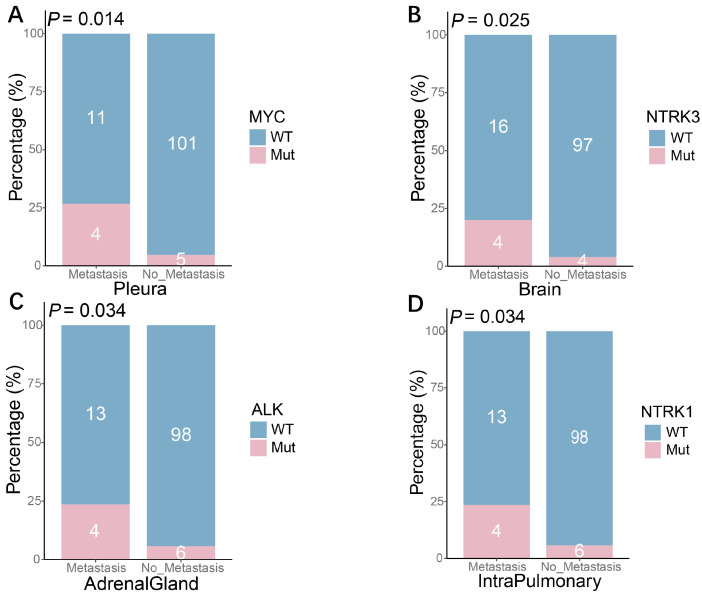
Correlation of gene mutations with specific metastatic sites in small-cell lung cancer: (**A**) Higher frequency of *MYC* mutations in patients with pleural metastasis. (**B**) Higher frequency of *NTRK3* mutations in patients with brain metastasis. (**C**) Higher frequency of *ALK* mutations in patients with adrenal metastasis. (**D**) Higher frequency of *NTRK1* mutations in patients with intrapulmonary metastasis.

Metastatic spread is a hallmark of SCLC and a key determinant of its poor prognosis; however, the specific genetic alterations that underlie this aggressive behavior remain poorly defined. To explore this, we examined potential associations between somatic mutations and specific metastatic patterns. Our analysis revealed several notable associations: *MYC* mutations were more frequent in patients with pleural metastases (26.7% vs. 4.7%; *p* = 0.014). Similarly, *NTRK3* mutations were observed more commonly in cases with brain metastases (20.0% vs. 4.0%; *p* = 0.025), while *ALK* and *NTRK1* mutations were associated with adrenal (23.5% vs. 5.8%; *p* = 0.034) and intra-pulmonary metastases (23.5% vs. 5.8%; *p* = 0.034), respectively. These results indicate that mutations in *MYC*, *NTRK3*, *ALK*, and *NTRK1* are significantly associated with distinct metastatic patterns in SCLC, suggesting their potential involvement in organ-specific dissemination (Figure 5).

**Figure 6:**
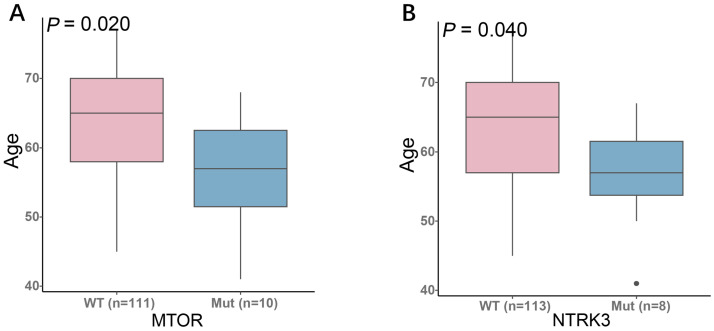
Gene mutations associated with younger age in small-cell lung cancer: (**A**) Higher frequency of *MTOR* mutations in younger patients. (**B**) Higher frequency of *NTRK3* mutations in younger patients.

**Figure 7:**
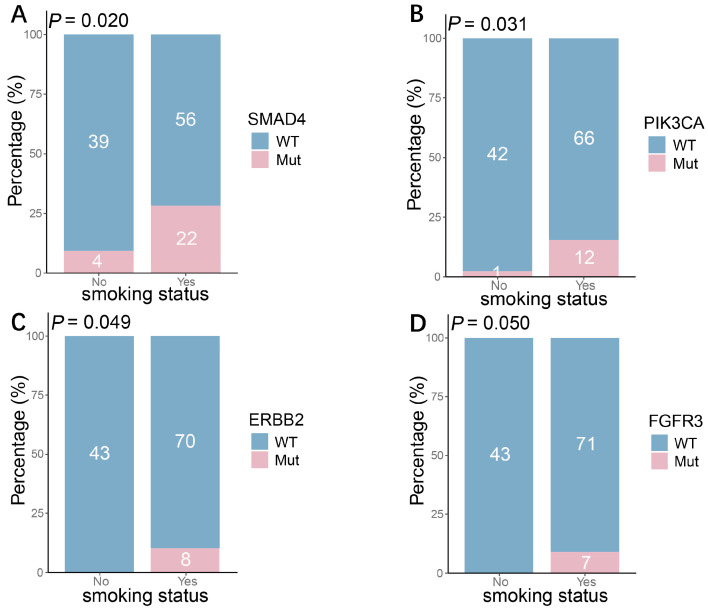
Association of gene mutations with smoking status in SCLC: (**A**–**D**) Mutation frequencies of *SMAD4* (**A**), *PIK3CA* (**B**), *ERBB2* (**C**), and *FGFR3* (**D**) were all significantly higher in smokers than in non-smokers.

We next investigated the associations between gene mutations and other clinical features. Analysis by age group revealed that mutations in *MTOR* and *NTRK3* were significantly enriched in younger patients, suggesting a potential age-related molecular pathogenesis in SCLC (Figure 6). Furthermore, we evaluated the relationship between smoking history and the genomic landscape. Smokers exhibited significantly higher mutation frequencies in *SMAD4* (*p* = 0.020), *PIK3CA* (*p* = 0.031), *ERBB2* (*p* = 0.049), and *FGFR3* (*p* = 0.050) compared to non-smokers. Notably, *SMAD4* mutations were observed in 75% of smokers, highlighting a strong association with smoking history (Figure 7).

## 4. Discussion

SCLC represents the most aggressive subtype of lung cancer, characterized by rapid progression, high relapse rates, and an exceedingly poor prognosis, with a five-year overall survival of only 10% [15]. Approximately 70% of SCLC cases are diagnosed as ES-SCLC, with the majority of patients presenting with systemic metastasis at initial diagnosis. For decades, platinum-based doublet chemotherapy has remained the standard first-line treatment for ES-SCLC. Although initial responses are often favorable, patients frequently develop rapid resistance, leading to disease progression. The introduction of ICIs has recently transformed the treatment landscape. Currently, PD-L1 inhibitors combined with platinum and etoposide chemotherapy have become the first-line standard of care for ES-SCLC [16]. Landmark trials such as IMpower133 and CASPIAN established the survival benefit of this approach, demonstrating significant improvements in both PFS and OS with the addition of atezolizumab or durvalumab to chemotherapy, respectively [2,6]. These consistent findings underscore the role of immunotherapy as an effective strategy for improving outcomes in ES-SCLC [17]. Building on the efficacy established in these clinical trials, this study further evaluates the real-world clinical performance of CIT using real-world data.

In this real-world cohort, we compared the efficacy of first-line CT (*n* = 55) versus CIT (*n* = 115) in 170 patients with SCLC. The median PFS was 14.5 months (95% CI: 10.1–18.5) in the CT group and 15.0 months (95% CI: 13.5–17.3) in the CIT group. Although a numerical extension in median PFS was observed with CIT, the difference was not statistically significant. This aligns with several prior real-world analyses that also reported no significant PFS or OS difference between the two regimens [18]. The attenuated PFS benefit of CIT in real-world settings may be influenced by patient heterogeneity, variations in treatment patterns, or limited follow-up duration. Notably, however, the CIT group demonstrated superior objective and disease control response rates (ORR, DCR), suggesting a more pronounced initial antitumor effect. The failure of this response advantage to translate into a significant PFS benefit may involve complex factors, including baseline patient characteristics, intrinsic tumor biology, and adaptive changes in the tumor microenvironment, or mechanisms of immune resistance.

Furthermore, univariate Cox regression analysis identified liver metastasis as an independent adverse prognostic factor in SCLC, a finding consistent with multiple large-scale retrospective studies and meta-analyses, reaffirming the clinical significance of liver metastasis as a strong negative prognostic marker [19,20]. Notably, our study also found adrenal metastasis to be an independent prognostic factor. However, evidence regarding the prognostic value of adrenal metastasis remains relatively limited, and the underlying biological mechanisms are not yet fully understood, warranting further investigation.

We performed a systematic genomic analysis to identify co-occurring and mutually exclusive mutation patterns in SCLC, which revealed significant co-alterations among several key genes. For instance, *PIK3CA* mutations frequently co-occurred with alterations in *PTEN*, *ATM*, *ERBB2*, and *BRCA1*. These genetic events were not randomly distributed but were highly enriched within several core signaling pathways, most notably revealing a coordinated activation pattern in the PI3K/AKT/mTOR pathway. Within this pathway, *PIK3CA* appears to function as a hub gene, concurrently altered alongside multiple upstream receptor tyrosine kinases (e.g., *ERBB2*, *FGFR3*), intra-pathway components (e.g., *AKT1*, *MTOR*), and negative regulators (e.g., *PTEN*).

Both *PIK3CA* activating mutations and *PTEN* loss are well-established driver events in tumor progression. As a key negative regulator of the PI3K-AKT-mTOR signaling pathway [12], the co-occurrence of *PTEN* loss with *PIK3CA* mutation may reflect a “dual activation” mechanism that synergistically amplifies oncogenic signaling output. On one hand, *PIK3CA*-activating mutations or RTK amplification enhance forward signaling; on the other hand, *PTEN* loss removes intrinsic inhibitory control, thereby cooperatively driving hyperactivation of the pathway and promoting tumor cell proliferation, survival, and metabolic reprogramming. These findings also provide a new perspective for understanding therapy resistance in SCLC. Alterations in the *PIK3CA/AKT/PTEN* pathway have been linked to resistance to targeted agents such as osimertinib in NSCLC and other malignancies [8,9]. Our data suggest that a similar cooperative pathway activation mechanism may contribute to the development of resistance to chemotherapy or immunotherapy in SCLC, warranting further functional studies for validation.

Additionally, our analysis revealed significant crosstalk between the DDR pathway and RTK/PI3K signaling. Specifically, we identified frequent co-alterations involving key gene pairs, including *PIK3CA* with *ATM* or *BRCA1*, *AKT1* with *BRCA1*, and *ERBB2* with *ATM*. This co-occurrence pattern suggests a potential functional link between growth factor pathway activation and genomic instability in a subset of SCLC cases. As a core component of the DDR pathway, the role of *ATM* in related malignancies has garnered increasing attention. Supporting this notion, a study in bladder cancer demonstrated that a classifier based on *TP53/PIK3CA/ATM* mutation status could predict patient responses to ICI therapy [21].

Of particular note was the observed co-occurrence of *BRCA1* and *BRCA2* mutations, strongly suggesting the presence of HRD. HRD status is not only associated with genomic instability but also serves as an important biomarker predicting sensitivity to PARP inhibitors and platinum-based chemotherapy [22]. Previous studies have indicated that the combination of PARP inhibitors and immune checkpoint inhibitors demonstrates promising efficacy and tolerability in patients with advanced solid tumors [23]. Therefore, SCLC patients with HRD features may represent a potential beneficiary population for PARP inhibitor-targeted therapy.

On the other hand, our study revealed a significant pattern of mutual exclusivity between *STK11* and *RB1* mutations, suggesting distinct biological dependencies in SCLC pathogenesis. This mutual exclusion may reflect divergent dependencies of SCLC cells on specific signaling pathways: tumors with *RB1* inactivation might rely on *STK11*-mediated metabolic regulation, and vice versa. This phenomenon aligns with observations in large cell neuroendocrine carcinoma (LCNEC), where *RB1* mutations and *STK11* alterations rarely co-occur [24]. Furthermore, emerging evidence in NSCLC links *STK11* mutations to immunotherapy resistance, potentially via metabolic reprogramming that fosters an immunosuppressive microenvironment [25,26]. These insights reinforce the functional significance of *STK11* beyond metabolism, implicating it in treatment evasion and tumor aggressiveness.

## 5. Conclusions

In summary, our integrative analysis of co-occurring and mutually exclusive genetic alterations in SCLC reveals an interactive molecular network that extends beyond the canonical *TP53/RB1* inactivation paradigm. These findings deepen the mechanistic understanding of SCLC biology and provide a framework for developing targeted therapeutic approaches. Promising strategies include combined inhibition of the PI3K/AKT/mTOR pathway, PARP inhibitor application guided by HRD status, and subtype-specific treatments based on *STK11/RB1* mutual exclusivity. Future studies should explore the functional and clinical relevance of these patterns for precision medicine applications in SCLC.

Our analysis of the real-world SCLC cohort revealed significant associations between specific genetic alterations and distinct patterns of metastatic spread. Notably, we observed that *NTRK3* mutations were significantly correlated with brain metastases, suggesting a potential role for the *NTRK* signaling pathway in the development of SCLC brain metastasis. It is important to emphasize that the *NTRK3* alterations identified in this cohort were primarily missense mutations, not the *NTRK* gene fusions targeted by approved TRK inhibitors such as larotrectinib or entrectinib [27]. While these inhibitors have demonstrated high response rates in various *NTRK* fusion-positive cancers [28,29], their efficacy against *NTRK3* missense mutations in SCLC remains to be investigated. Furthermore, significant correlations were observed between *MYC* mutations and pleural metastasis, *ALK* mutations and adrenal metastasis, and *NTRK1* mutations and intrapulmonary metastasis, indicating that specific genetic alterations may drive the tropism of tumor cells to particular organs.

Our study also uncovered a significant association between smoking history and specific mutational patterns. Mutations in genes such as *SMAD4*, *PIK3CA*, *ERBB2*, and *FGFR3* were significantly enriched in smokers, with *SMAD4* mutations being particularly prominent. This aligns with a report from the Japanese National Cancer Center, which indicated that passive smoking induces numerous subclonal mutations with an APOBEC signature, including hotspot mutations in *SMAD4* [30]. Research in NSCLC has shown that smoking can induce epigenetic reprogramming of the TGF-β/SMAD3 pathway and that mutations in *KRAS* and *SMAD4* are more frequent in smokers [31,32]. The high frequency of *SMAD4* mutations is of particular biological relevance. As a core tumor suppressor within the TGF-β signaling pathway [33], its inactivation might promote epithelial–mesenchymal transition (EMT) and contribute to an immunosuppressive tumor microenvironment, thereby potentially enhancing tumor invasiveness. Conversely, mutations in *MTOR* and *NTRK3* were more frequent in younger patients, suggesting that they may define a molecular subtype associated with age, whose pathogenesis might be more related to endogenous developmental or metabolic pathway dysregulation rather than direct tobacco exposure. This divergence in molecular backgrounds between smoking-associated mutations and those enriched in younger patients further underscores the significant molecular heterogeneity of SCLC across different clinical contexts.

This study is characterized by its analysis of real-world clinical treatment responses and genomic data, providing evidence that SCLC exhibits heterogeneity at both clinical and molecular levels. Yet, the study has several limitations, such as a retrospective design, potential selection bias, and an incomplete overall survival analysis caused by missing follow-up data. Future studies with larger, prospective cohorts are needed to validate these results and to further explore the molecular mechanisms, including associations between protein expression and genomic alterations, and their causal links to treatment outcomes.

## Figures and Tables

**Table 1:** Baseline patient demographics and clinical characteristics by treatment group.

	Overall (*n* = 170)	Chemotherapy Group (*n* = 55, %)	Chemoimmunotherapy (*n* = 115, %)	*p* Value
Gender				0.664
Male	139	46 (83.6)	93 (80.9)	
Female	31	9 (16.4)	22 (19.1)	
Age				0.553
Median, range (years)	63.5 (62, 66)	65 (62, 67)	63 (60, 66)	
<65	86	26 (47.3)	60 (52.2)	
≥65	84	29 (52.7)	55 (47.8)	
SCLC type				0.773
Central	123	39 (70.9)	84 (73.0)	
Peripheral	47	16 (29.1)	31 (27.0)	
Clinical stage				0.966
IIIa	4	2 (3.6)	2 (1.7)	
IIIb	19	9 (16.3)	10 (8.7)	
IIIc	23	4 (7.2)	19 (16.5)	
IVa	46	14 (25.5)	32 (27.8)	
IVb	78	26 (47.3)	52 (45.2)	
Smoking history				0.463
Ever	114	39 (70.9)	75 (65.2)	
Never	56	16 (29.1)	40 (34.8)	
Specimen site				0.558
Lung	81	27 (49.1)	54 (47)	
Lymph node	82	27 (49.1)	55 (47.8)	
Other ^a^	7	1 (1.8)	6 (5.2)	
ECOG PS				0.846
0	5	1	4	
1	154	50	104	
2	2	1	1	
NA	9	3	6	
TPS of PD-L1				0.62
<1%	128	38 (69.1)	90 (78.3)	
1–49%	17	9 (16.3)	8 (67)	
≥50%	0	0 (0)	0 (0)	
Unknown	25	8 (14.5)	17 (14.7)	
Immunohistochemistry				
CD56	167	54 (98.2)	113 (98.3)	0.693
CK	167	55 (100)	112 (97.4)	0.307
TTF1	151	50 (90.9)	101 (87.8)	0.033
SYN	86	34 (94.4)	52 (96.3)	0.071
ChgA	26	10 (30.3)	16 (0.32)	0.129
INSM1	75	28 (84.8)	47 (97.9)	0.006
Ki-67	78.3	77.7	79.1	0.416
Metastatic sites				
Bone	55	13 (23.6)	42 (36.5)	0.094
Liver	33	10 (18.2)	23 (20)	0.781
Brain	30	15 (27.3)	15 (13)	0.023
lymph nodes	24	7 (12.7)	17 (14.8)	0.721
Adrenal	24	10 (18.2)	14 (12.2)	0.295
Intrapulmonary	22	10 (18.2)	12 (10.4)	0.161
Pleura	24	10 (18.2)	14 (12.2)	0.374
Other	18	5 (9)	13 (11.3)	0.663
Treatment				
PD-1 inhibitor	16	0 (0)	16 (13.9)	-
PD-L1 inhibitor	97	0 (0)	97 (84.3)	
Progression status				0.374
Yes	119	41 (74.5)	78 (67.8)	
No	51	14 (25.5)	37 (32.2)	
Best response				0.011
PR	119	31 (56.4)	88 (76.5)	
SD	40	18 (32.7)	22 (19.1)	
PD	11	6 (10.9)	5 (4.4)	

PD-L1, programmed cell death ligand-1; PD-1, programmed cell death protein 1; TPS, tumor proportion score; CD56, cluster of differentiation 56; CK, cytokeratin; TTF1, thyroid transcription factor-1; SYN, synaptophysin; ChgA, chromogranin A; INSM1, insulinoma-associated protein 1. PR, partial response; SD, stable disease; PD, progressive disease. A *t*-test was used for the difference in age; the chi-square test for the difference in gender, SCLC type, smoking history, specimen site, metastatic sites, treatment, progression status, and immunohistochemistry; and the Wilcoxon rank-sum test for the difference in clinical stage, TPS of PD-L1, and best response. ^a^ Includes hydrothorax, pleura, chest wall mass, and liver.

**Table 2:** Comparison of efficacy between the CT and CIT groups.

Therapeutic Efficacy	Chemotherapy Group (*n* = 55)	Chemoimmunotherapy (*n* = 115)	*p* Value
PR, *n* (%)	31 (56.4)	88 (76.5)	0.011
SD, *n* (%)	18 (32.7)	22 (19.1)	
PD, *n* (%)	6 (10.9)	5 (4.4)	
ORR, (%) (95% CI)	56.4 (43.3, 68.6)	76.5 (67.9, 83.3)	0.007
DCR, (%) (95% CI)	89.1 (78.2, 94.9)	95.7 (90.2, 98.1)	0.10

PR, partial response; SD, stable disease; PD, progressive disease; ORR, objective response rate; DCR, disease control rate.

**Table 3:** Univariate analyses of progression-free survival in ES-SCLC patients.

Characteristics	HR	95% CI	*p* Value
Age, years ≥65 vs. <65	0.97	(0.68–1.39)	0.86
Gender Male vs. Female	1.41	(0.86–2.32)	0.17
Smoking history Yes vs. No	1.3	(0.88–1.94)	0.19
Subtyping central vs. peripheral	0.81	(0.54–1.21)	0.31
Stage IV vs. III	1.34	(0.89–2.02)	0.16
Metastatic sites			
Bone	1.33	(0.9–1.95)	0.15
Liver	1.68	(1.10–2.58)	0.02
Brain	1.22	(0.77–1.92)	0.4
lymph nodes	1.21	(0.74–1.97)	0.45
Adrenal	1.68	(1.04–2.72)	0.04
Intrapulmonary	1.01	(0.77–1.32)	0.96
Pleura	1.1	(0.81–1.48)	0.55
TPS of PD-L1 1–49% vs. <1%	1.14	(0.65–1.99)	0.66
Treatment mode CIT vs. CT	0.83	(0.57–1.21)	0.33

**Table 4:** Multivariate analyses of progression-free survival in ES-SCLC patients.

Characteristics	HR	95% CI	*p* Value
Age, years ≥65 vs. <65	1.1	(0.75–1.62)	0.64
Gender Male vs. Female	1.13	(0.55–2.35)	0.74
Smoking history Yes vs. No	1.26	(0.70–2.28)	0.45
Subtyping central vs. peripheral	0.74	(0.48–1.13)	0.16
Stage IV vs. III	1.1	(0.61–1.94)	0.77
Metastasis			
Bone	1.02	(0.61–1.71)	0.94
Liver	1.56	(0.89–2.73)	0.12
Brain	1.02	(0.6–1.74)	0.94
lymph nodes	1.13	(0.63–2.0)	0.69
Adrenal	1.84	(1.06–3.22)	0.03
Intrapulmonary	0.751	(0.40–1.42)	0.38
Pleura	0.79	(0.40–1.56)	0.5
TPS of PD-L1 1–49% vs. <1%	0.99	(0.54–1.82)	0.98
Treatment mode CIT vs. CT	0.9	(0.58–1.36)	0.6

## Data Availability

The data presented in this study are available upon request from the corresponding author.
